# Conditional correlation network data from the financial sector

**DOI:** 10.1016/j.dib.2022.107858

**Published:** 2022-01-21

**Authors:** Paul Borochin, Stephen Rush

**Affiliations:** a University of Miami, Coral Gables, FL 33146, USA; b Bowling Green State University, Bowling Green, OH 43403, USA

**Keywords:** Systemic risk, Network analysis, Informed trading

## Abstract

This data set contains rolling conditional correlation networks estimated from stock returns and the volume synchronized probability of informed trading. Only the largest 104 financial firms are included for the period of 1996 through 2012. The data was used to analyze banking sector systemic risk in Borochin and Rush (2022)[1].

## Specifications Table

**Table:** 

Subject	Financial Markets and Institutions
Specific subject area	Financial Market Stability
Type of data	Table
How data were acquired	The data was produced using the following software: bnlearn software package for R [2] python programming language R programming language
Data format	Filtered
Parameters for data collection	We combine the New York Stock Exchange Trade and Quote (NYSE TAQ), the Center for Research in Security Prices (CRSP), and OptionMetrics datasets. We limit the sample to financial firms and estimate the network structure.
Description of data collection	We match the NYSE TAQ data set to CRSP common equity. We retain firms with Standard Industrial Classification (SIC) Industry codes from 6000 to 6800 before merging with OptionMetrics. We eliminate firms with prices less than $5 per share, daily trading volume less than 1000 shares, and market capitalization of less than $100mm. We form an hourly panel of VPIN and stock returns, then estimate daily conditional correlation networks using the bnlearn software.
Data source location	Bowling Green State University, Bowling Green, OH 43403, USA
	Primary data sources: New York Stock Exchange Trade and Quote database, the Center for Research in Security Prices (CRSP) database, and OptionMetrics database
Data accessibility	Repository name: Mendeley Data Data identification number: 10.17632/pns8jrrc7h.1 Direct URL to data: https://doi.org/10.17632/pns8jrrc7h.1
Related research article	Borochin, P., S. Rush, Information Networks in the Financial Sector and Systemic Risk, Journal of Banking and Finance. 134 (2022). https://doi.org/10.1016/j.jbankfin.2021.106327

## Value of the Data

• With current technology, conditional correlation networks estimated from the Volume Synchronized Probability of Informed Trading measure as well as intraday stock returns take months of continuous computer cluster time to estimate from the raw Trade and Quote data. Those without large computing resources will benefit from this data.
• Estimates of the connections between financial firms are useful to academics, practitioners, and regulatory authorities interested in monitoring the stability of the financial system.
• This data may be combined with other relevant data on the financial system to improve our measurement of financial stability.

## 1 Data Description

The data set contains two zip files: “Returns” for return networks and “VPIN” for VPIN networks. Each zip file contains 4271 R binary files using the “RData” extension for a total of 8542 files. The file name format is “NetVPIN_YEAR_DAY.RData” where the day is the numbered trading day from the beginning of that year. Example: “NetVPIN_1996_1.RData” After loading the binary file in R, there will be a single R object “net” of class “bn” from the bnlearn package. This object contains the conditional correlation network estimated over the past 100 trading days

## 2 Experimental Design, Materials and Methods

We construct the intital data set by matching the NYSE TAQ data set to CRSP common equity and find 15,340 unique CUSIPs. Next, we use SIC Industry codes from 6000 to 6800 to select 3891 financial firms from the combined TAQ and CRSP data set. Merging with OptionMetrics leaves us with 302 firms. We eliminate firms with prices less than $5 per share, daily trading volume less than 1000 shares, and market capitalization of less than $100mm. The resulting sample contains 104 unique financial firms from 1996 through 2012.

We calculate the Volume Synchronized Probability of Informed Trading (VPIN) according to Easley, López de Prado, and O’Hara (2012)[2] using bulk volume classification. Each firm has a unique volume bucket size and observation frequency depends on individual trading volume on each day. The volume clock increment for each firm is the average daily volume divided by 50. This volume clock increment is unique for each stock year. This permits variation over time and across firms. We use bulk volume classification to assign a percentage of each volume bucket as sell or buy order flow. Buyer initiated volume $\left(V_{t}^{B}\right)$ is calculated as follows:

(1) $$V_{t}^{B}=V_{t}\times \phi \left(\frac{\Delta price_{t}}{\sigma_{\Delta price}}\right)$$

where $V_{t}$ is the total volume in a volume bar, $\Delta price_{t}$ is the change in price from the beginning of the volume bar to the end of the volume bar, $\sigma_{\Delta price}$ is the standard deviation of price changes over the last 50 volume bars which represents an average day’s volume, and ϕ is the cumulative standard normal distribution. The entire volume bar is divided between seller initiated volume and buyer initiated volume. Seller initiated volume, $V_{t}^{S}$, is the remaining volume in the volume bar $V_{t}^{S}=V_{t}-V_{t}^{B}$. We then calculate VPIN as:

(2) $$VPIN_{t}=\frac{E[|V_{t}^{B}-V_{t}^{S}|]}{V_{t}}$$

We use a time weighted average for aggregation to the hourly frequency and do not allow prior observations to continue beyond the hour after they occur. Within each hour, we weight each observation by either the minutes before the next observation or the end of the hour divided by sixty. Observations last a maximum of two hours before being replaced by a null value if it is not replaced by a more recent observation.

We use a rolling 100 trading day window of hourly VPIN data to estimate the conditional correlation network of the VPIN measure. The network structure is estimated following Scutari (2010)[3] with a score-based hill climbing algorithm that optimizes the Bayesian Information Criterion (BIC) of the network. The BIC is defined as follows:

(3) $$BIC=\sum_{i=1}^{n}\log f_{x_{i}}\left(X_{i}|\Pi_{X_{i}}\right)-\frac{d}{2}\log n$$

where $X_{i}$ is each stock, $\Pi_{X_{i}}$ is the subset of X directly connected to $X_{i}$, n is the number of stocks, d is the number of parameters in the joint density function, and $f_{X}$ is the joint density function. The factorization of $f_{X}$ is:

(4) $$f_{X}\left(X\right)=\prod_{i=1}^{p}f_{X_{i}}\left(X_{i}|\Pi_{X_{i}}\right)$$

Here, the additional parameter p, is the number of stocks with edges.

The bnlearn software begins with a random network structure, calculates the BIC, adds or deletes an edge, recalculates the BIC, and keeps the change and replaces the prior best score or tries again with a different edge. The process is repeated until the marginal change in BIC approaches zero. The algorithm does not guarantee and global maximum so we randomly restart the process 10 times to avoid local maxima. Initial tests with data sub-samples revealed no changes in network structure using 5 random restarts. We increased random restarts to 10 in the event that some time periods were dramatically different than the sub-sample.

The estimated network structure is composed of nodes that represent firms and edges that represent conditional correlation. If a significant portion of the variation in a single firm is explained by its neighbors, an edge is drawn from the neighbors to the firm. The network estimation finds the optimal neighbors and number of neighbors for each node to maximize the individual firm variation explained by the neighbors of each node. This network is estimated for both hourly stock returns and hourly observations of the VPIN measure.

## CRediT authorship contribution statement

**Paul Borochin:** Supervision, Project administration, Resources. **Stephen Rush:** Conceptualization, Methodology, Software, Formal analysis, Resources, Data curation, Writing – original draft.

## Declaration of Competing Interest

The authors declare that they have no known competing financial interests or personal relationships which have, or could be perceived to have, influenced the work reported in this article.

## References

[1] Borochin P., Rush S. (2022). Information networks in the financial sector and systemic risk. Journal of Banking and Finance.

[2] Easley D., López de Prado M.M., O’Hara M. (2012). Flow toxicity and liquidity in a high-frequency world. Review of Financial Studies.

[3] Scutari M. (2010). Learning bayesian networks with the bnlearn r package. Journal of Statistical Software.

